# Antimicrobial screening of silver nanoparticles synthesized by marine cyanobacterium *Phormidium formosum*

**Published:** 2020-06

**Authors:** Reham G. Elkomy

**Affiliations:** Department of Marine Environment, Marine Biotechnology Lab, National Institute of Oceanography and Fisheries (NIOF), Alexandria, Egypt

**Keywords:** Antimicrobial activity, Silver nanoparticles, Biosynthesis, Marine cyanobacteria

## Abstract

**Background and Objectives:**

Nanoparticles are widely used in various fields such as electronics, cosmetics, water purification, biomedical and biotechnology. Biosynthesis of nanoparticles using biological agents have gained much attention in the area of nanotechnology in the last few decades because of cost effective, non-toxic, and eco-friendly. Algae have been used to reduce metal ions and subsequently for the biosynthesis of nanoparticles.

**Materials and Methods:**

Silver nanoparticles (AgNPs) have been biosynthesized by *Phormidium formosum* isolated from Mediterranean Sea coast Egypt in an aqueous system. An aqueous solution of silver ions was treated with alive biomass of *P. formousm* for the formation of AgNPs. The physio-chemical properties of synthesized silver nanoparticles were studied using analytical techniques such as UV-Vis spectrophotometer, transmission electron microscopy (TEM), and Fourier Transform Infrared Spectroscopy (FTIR). The antimicrobial effect of synthesized silver nanoparticles was also tested on several microorganisms by measuring the inhibition zone.

**Results:**

These nanoparticles showed an absorption peak at λmax 437 nm in the UV-visible spectrum, corresponding to the Surface Plasmon Resonance of AgNPs. The transmission electron micrographs of nanoparticles in an aqueous solution showed production of silver nanoparticles synthesized by *P. formosum*. The obtained AgNPs are spherical in shape with a size ranging from 1.83 nm to 26.15nm. The Fourier transmittance infrared spectrum (FTIR) confirms the presence of bio component in alive biomass of *P. formosum* which was responsible for the nanoparticles synthesis. The antimicrobial test revealed that AgNPs synthesized by *P. formosum* is capable to inhibit the growth of microorganisms.

**Conclusion:**

The results confirmed that AgNPs can act as a powerful antimicrobial agent against fish and human pathogens.

## INTRODUCTION

The novel properties of chemical nanoparticles are highly sought after in a number of new and existing industries. In recent years, nanotechnology is an escalating field of modern research involving in synthesis design, characterization, production, and application of structures, devices, and systems by controlling shape and size at the nanometer scale (1–2). Biological methods for synthesizing nanoparticles have shown better results compared to chemical and physical techniques due to time consumption, slow kinetics (3–4) low cost (5) and most importantly it is eco-friendlier compared to the chemical and physical techniques (6). Algae, fungi, bacteria; as well as biomolecules such as proteins, amino acids, carbohydrates and sugars could be used for biosynthesis of nanoparticles (7). Many centuries ago silver particles were used as an antimicrobial substance. Oligo dynamic effects of silver enables the binding of silver ions to the reactive sites of the bacterial cells, resulting in precipitation and inactivation of the organisms (8). As a result, nano silver particles based antiseptics are mainly in use nowadays (9). Algae used in the biosynthesis of nanoparticles have recently attracted the attention of many scientific researches due to their rapid growth, to their biomass formation and to their different biological activities by nature. Extensive work has been conducted on different types of algae mainly *Spirulina platensis* for the biosynthesis of nanoparticles. So far, Mohammed et al. and Ahmed et al. (10–11) have used cyanobacteria for the synthesis of silver nanoparticles; they reported good antibacterial activity against some human pathogenic bacteria.

In this study, a marine diatom was used to reduce silver nitrate into nano-sized silver particles. The biosynthesized silver nanoparticles have been characterized through UV-Vis spectroscopy, the Transmission Electron Microscopy (TEM) and Fourier Transform Infrared Spectroscopy (FTIR). Furthermore, biosynthesized silver nanoparticles (AgNPs) were then tested for antimicrobial activity using Gram-positive and Gram-negative bacteria.

## MATERIALS AND METHODS

### Preparation of algal biomass.

The algal strains *Phormidium formosum*, was isolated from Eastern harbor) in the Mediterranean coast of Egypt. Samples were grown in 500 ml culture flasks containing F/2 medium (12–13). The growth potential of alga was maintained through regular sub culturing techniques, under laboratory conditions at pH 7, 28°C, and illuminated with cool white fluorescent lamps at an intensity 100 μmol photons m^−2^ s^−1^. The algal cultures were supplied with dry air (14) to provide CO_2_ necessary for photosynthesis, to prevent the settling of cells bottom of the containers and maintain the alga in suspension without mechanical stress (15). The algal strains were harvested at their exponential phase of growth which is 8^th^ day. Harvesting took place by centrifugation at 4000 rpm for 15 min. The isolated strains were identified according to the published protocols (16–18).

### Development of silver nanoparticles.

The AgNPs were prepared by taking 5 g of thoroughly washed *Phormidium formosum* biomass from an exponential growth phase in a 250 mL Erlenmeyer flask with 100 mL of 1 mM aqueous AgNO_3_ solution (pH 7) for 24 h (19). The entire process of the reduction of metal ions to nanoparticles was carried out at 28°C. After complete reduction, the synthesized medium was centrifuged at 1200 rpm for 30 min. The pallet was collected and dried in an oven at 45°C. The biosynthesis reaction was achieved without using any catalytic chemicals and polymers as a stabilizing and capping agent (20).

### UV-vis spectra analysis.

The UV-vis spectra of silver nanoparticles were recorded by spectrophotometer (Thermos Spintronic) at resolution of 1.0 nm at wave-length 300 nm to 600 nm.

### TEM analysis of silver nanoparticles.

Transmission Electron Microscopic (TEM) analysis was done using a TEM, JEM-1200EX (JEOL Ltd., Japan). 3 μL of the sample solution was placed on the carbon coated copper grid, making a thin film of sample on the grid, and an extra sample was removed using a cone of blotting paper, and kept in a grid box, sequentially.

### Fourier transform infrared spectroscopy (FTIR).

Further synthesized silver nanoparticles were prepared in thin pellets using potassium bromide (KBr) for FTIR analysis. IR spectra were obtained from PERKIN Elmer model at the resolution of 1 cm^−1^ in the range of 4000 to 450 cm^−1^.

### Antimicrobial activity of silver nanoparticles.

Antimicrobial activity of silver nanoparticles synthesized from marine cyanobacterium *Phormidium formousum* was carried out by the disc diffusion method. *Serratia marcescens, Salmonella* spp., *Vibrio* spp., *Aeromonas hydrophilic, Pseudomonas aeruginosa, Escherichia coli* and *Proteus* spp. (Gram-negative bacteria); *Staphylococcus aureus, Micrococus luteus* and *Enterococcus faecalis* (Gram-positive bacteria); *Candida albicans* (fungus) were used in the antimicrobial susceptibility testing. Deionized water was used as the negative control. Bacterial cultures were spread equally onto the agar plate. The prepared sterile paper discs containing 10 μl of concentrations of silver nanoparticles and water extract of *P. formosum* (1mg per disc) was placed onto the bacterial cultured agar plateon sterilized paper disc (0.5 cm diameter). A pre diffusion for 2 h was carried out at 4°C before incubation (21). Inhibition zones were measured after 24 h incubation period at 37°C for microorganisms. Meanwhile, cefprozil and polymixin for Gram-negative and Gram-positive bacterial and fungal species were used respectively, as positive controls.

### Statistical analysis.

All measurements were carried out in triplicate. Statistical analyses were performed using one-way Analysis of Variance (ANOVA), and the significance of the difference between means was determined by Duncan’s multiple range tests. Differences at P<0.05 were considered statistically significant. The results were presented as mean values (± SD, Standard Deviations) (22).

## RESULTS

### Characterization of AgNPs by UV-visible spectroscopy.

**Fig. 1.**
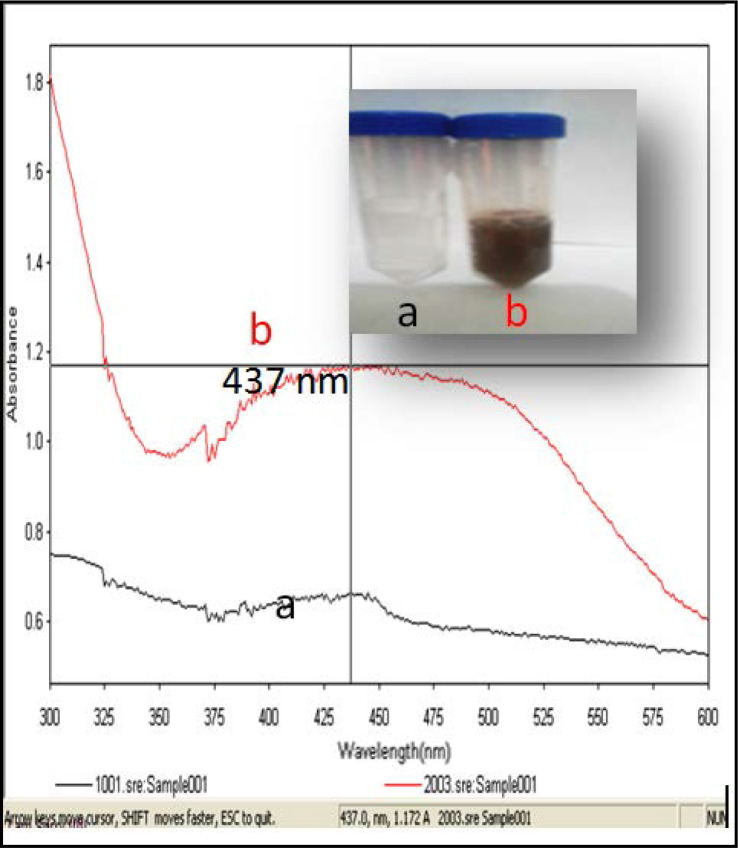
The conversion of nitrate (a) to nano silver (b) by *P. formosum* biomass. The images show the colour changes before (a) and when (b) the method of reduction of ionic silver (Ag+) to AgNPs and UV-Visible spectra recorded after the reaction of 1 mM silver nitrate answer (a) with five g *P. formousoum* wet biomass at hydrogen ion concentration seven and twenty-five °C and formation of AgNPs (b).

Colorless solution of silver nitrate turned into purplish brown in an hour after the addition of algal culture. Fig. 1 shows the color change of silver nanoparticles from clear to brown as an evident of AgNPs synthesized via *P. formosum*. Moreover, the UV-spectra provides strong evidence of nanoparticle formation. Light wavelength of 300–600 nm at UV-visible spectroscopy is commonly used to characterize various sizes of metal nanoparticles (23). Fig. 1 shows the absorbance spectrum at λmax = 437 nm while the absorption intensity 1.1, which indicates the formation of AgNPs in the solution.

### Characterization of AgNPs by TEM.

**Fig. 2.**
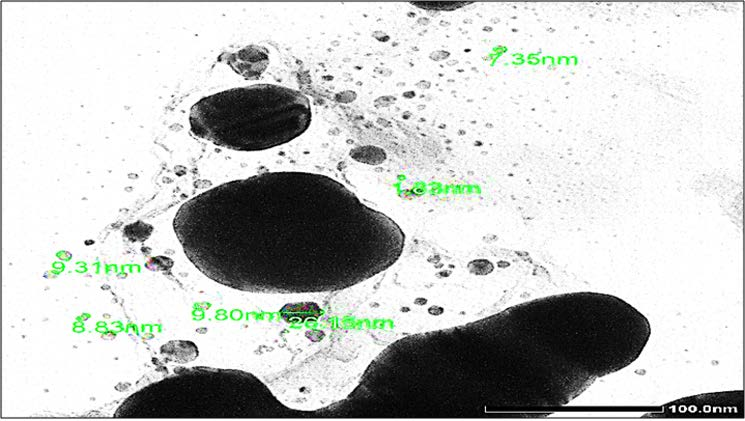
TEM image of developed AgNPs

The particle size distribution histogram for the SNPs determined from the TEM image is shown in Fig. 2. From this figure, it is clear that the particles, whose sizes range from 1 to 26 nm. TEM analysis showed that most particles had a size of ~9 nm. The average sizes of the particles depended on the organism used.

### FTIR spectra.

**Fig. 3.**
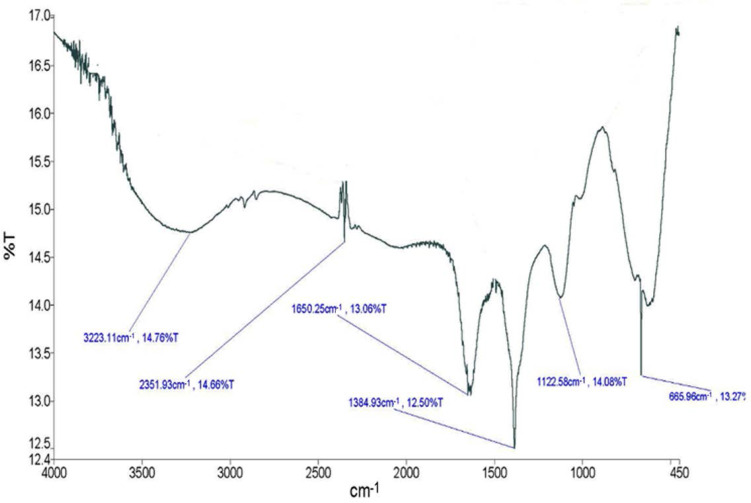
FTIR spectra of bio-synthesized AgNPs by *P. formosum*

The spectrum of FTIR evidently shows the bio fabrication of silver nanoparticles mediated by *P. formosum* at array of absorbance bands from 400 to 4000 cm^−1^. The results for *P. formosum* synthesized silver nanoparticles solution showed peaks at 3223.11, 2351.93, 1650.25, 1384.93, 1122.58 and 665.96 cm^−1^ as shown in Fig. 3. The peak and its attributions has summarized in Table 1. Display of strong broad O-H stretch carboxylic bands in the region 3223.11 cm^−1^ was observed.

**Table 1.** FTIR analysis of AgNPs synthesized by *P. formosum*

**Group frequency cm**^**−1**^	**Functional group assignment**
3223.11	O-H ( stretching-Carboxylic acids)
2351.93	O=C=O (stretch carbonic dioxide)
1650.25	C=C (stretch alkene)
1384.93	C-H (bend alkane)
1122.58	C-O (stretch aliphatic ether)
665.96	C-Cl or C-Br (stretch halo compounds)

### Antimicrobial activity of AgNPs.

**Fig. 4.**
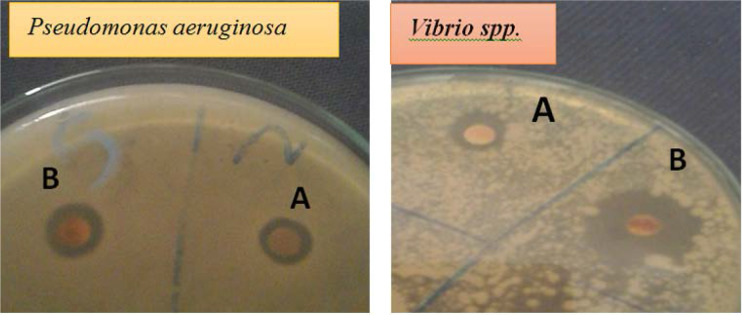
antagonistic effects of (A) *Phormidium formosum* extract by water and (B) AgNPs synthesized by *P. formosum* biomass against *Pseudomonas aeruginosa* and *Vibrio* spp.

The antimicrobial activity was evaluated as the diameters of the inhibition zones formed as a result of disc assay method in case of bacteria and fungi. The results of antimicrobial activity of AgNPs produced in this research are reported in Fig. 4 and Table 2. The antimicrobial test revealed that AgNPs synthesized by *P. formosum* is capable of inhibiting the growth of microorganism. Optimal inhibition zone of AgNPs at around 2.7 cm and 2.2 cm was observed for *Vibrio* spp. and *Staphylococcus aureus* respectively. The antimicrobial activity of AgNPs had more activity than water extract of *P. formosum*. The antimicrobial effect of AgNPs on *Staphylococcus aureus, Vibrio* spp., *Pseudomonas aeruginosa, Escherichia coli* and *Candida albicans* was more significant compared to standard antibiotic Cefprozil and Polymixin.

**Table 2.** Inhibition zone (cm) of silver nanoparticles synthesized by *P. formosum* and water extract of *P. formosum* against bacterial and fungal pathogens with antibiotic standard. Data are expressed as mean ± SE (n=3)

		**Diameter of inhibition zone (cm)**				

		**AgNPs synthesized by *P. formosum***	**Water extract by *P. formosum***	**Antibiotic as appositive control**		**Deionized water as negative control**

				**Cefprozil**	**Polymixin**	
Gram (+V) bacteria	*Staphylococcus aureus*	2.5 ± 0.03	1.8 ± 0.03	1.5 ± 0.05	2.0 ± 0.03	-
	*Micrococus luteus*	1.5 ± 0.03	0.8 ± 0.00	1.0 ± 0.03	1.5 ± 0.00	-
	*Enterococcus Faecalis*	1.3 ± 0.05	1.0 ± 0.05	1.5 ± 0.07	2.0 ± 0.08	-
Gram (− V) bacteria	*Serratia marcescens*	1.3 ± 0.03	1.0 ± 0.03	1.0 ± 0.03	1.5 ± 0.03	-
	*Salmonella* spp.	1.5 ± 0.03	1.1 ± 0.00	1.1 ± 0.00	1.5 ± 0.03	-
	*Vibrio* spp.	2.7 ± 0.03	1.2 ± 0.05	-	1.0 ± 0.03	-
	*Aeromonas hydrophila*	2.0 ± 0.05	1.0 ± 0.06	2.0 ± 0.05	2.3 ± 0.06	-
	*Pseudomonas aeruginosa*	1.5 ± 0.07	1.0 ± 0.00	1.0 ± 0.03	1.3 ± 0.00	-
	*Escherichia coli*	1.7 ± 0.00	1.0 ± 0.05	1.5 ± 0.00	1.6 ± 0.03	-
	*Proteus* spp.	1.5 ± 0.03	1.2 ± 0.03	1.1 ± 0.00	1.8 ± 0.00	-
Fungal species	*Candida albicans*	1.9 ± 0.03	1.3 ± 0.00	1.2 ± 0.02	1.6 ± 0.03	-

- =No inhibitory effect; width 0.1 to .08 cm = week activity; width 0.8 to 1.0 cm = moderate activity; width < 1.0 cm = strong activity

## DISCUSSION

In this study AgNO3 and *P. formosum* mixture were used to synthesize silver nanoparticles. The intensity of the color change increases with time. The change of color happens due to the formation of silver nanoparticles over time. The ability of enzymes responsible for the synthesis of silver nanoparticles increases under alkaline conditions (24). Similar to this study, the formation of silver nanoparticles had been indicated by the color change from a clear to brownish solution (25–26). The color change resulted from excitation of surface plasmon resonance (SPR) in the metal nanoparticles (27). Previous studies suggested that a usual silver nanoparticles SPR pattern is present at wavelength in the range of 400–480 nm (28). For example, Ahmed et al. observed that the band occurs at 400 nm and 402 nm for *Spirulina platensis* and *Nostoc* sp. (11). Silver reduction initiated through metabolites that was present in algae culture medium. The plasma bands are broad with an absorption tail in the longer wave length, which could be in principle due to size distribution of nanoparticles. The reduction of silver ions occurs through electron shuttle or through reducing agents released into solution by algal culture. Different wavelength readings indicate that AgNPs are formed in various particle sizes, shapes and surface properties. Therefore, such dissimilar physical formation of AgNPs is widely used as antimicrobial and antifungal agents in healthcare, food industry, textile coatings and electronic devices. TEM is powerful method to determine the size of nanoparticles (26). For example, it was observed that the smallest particles, 13 nm in diameter were formed with the biomass of *Spirulina* sp. while the largest ones were formed in *Limnothrix* sp. (29). The FTIR gives insights about the presence of functional groups in the synthesized silver nanoparticles in order to understand how they transform from simple inorganic silver nitrate to elemental silver due to the effect of different photo chemicals that might act in a simultaneous way as reducing, stabilizing and capping agent. These results revealed that the studied alga should be the origin of the bioactive groups which may be responsible for the reduction of metal ion to metal nanoparticles. Also, the used solvent may play an important role in the extraction of these compounds (30–31). The diameter of the inhibition zone depends on the species of bacteria and fungi (32). The mechanism of the bactericidal effect of SNPs is not very well-known. A suggested idea is AgNPs after penetration into the bacteria can inactivate their enzymes, generate hydrogen peroxide and cause bacterial cell death (33). The silver nanoparticles exhibited antibacterial activity in varying magnitudes. From the results obtained, it was noticed that the silver nanoparticles were more bactericidal against Gram-negative bacteria as compared to Gram-positive bacteria. Previous studies confirmed that silver nanoparticles were more bactericidal against Gram-negative bacteria (34–35). Optimal antibacterial effect was observed with Gram-negative bacteria since the thin cell wall with peptidoglycan allows for easier permeability when compared with thick cell wall made Gram-positive bacteria (36). Anyway, the results reveal a strong antimicrobial activity of AgNPs synthesized in this research.

## CONCLUSION

Synthesis of AgNPs using biological resources like marine cyanobacterium *Phormidium formosum* is a challenging alternative to chemical synthesis since this novel biogenic method is an eco-friendly method. The obtained data clearly indicate that alive biomass of *P. formosum* can be used as an effective capping as well as the reducing agent for the synthesis of AgNPs. Green synthesized silver nanoparticles are confirmed by a color change which was monitored quantitatively by UV-Vis spectroscopy at λmax 437 nm. Further characterization with TEM analysis shows the spherical in shape with a size ranging from 1.83 nm to 26.15 nm. FTIR showed the structure, the respective bands of the synthesized nanoparticles and the stretch of bonds. The synthesized AgNPs have shown antibacterial and antifungal activity with conventional antibiotics against a wide range of pathogenic bacteria which established their application in biomedicines. Moreover, this process could be easily scaled up for the industrial applications to increase the yield of the nanoparticles significantly, which undoubtedly would establish its commercial viability in medicine.
